# Comprehensive Management of Severe Mental Disorders: The Synergy between Multifamily Groups and Psychopharmacology

**DOI:** 10.1192/j.eurpsy.2025.2195

**Published:** 2025-08-26

**Authors:** R. Albillos Pérez, S. Castelao Almodóvar, R. González Núñez, A. Castelao Escobar, P. Sanz Sánchez, C. Alguacil Núñez

**Affiliations:** 1 Psiquiatría, Hospital Universitario Puerta de Hierro Majadahonda; 2 Psiquiatría, Hospital Universitario El Escorial, Madrid, Spain

## Abstract

**Introduction:**

The treatment of severe mental disorders benefits significantly from a multidisciplinary approach that integrates, among others, psychopharmacology and psychotherapy. The intervention with multifamily groups (MFG) shows multiple benefits for this patient profile. Its positive effects include reducing relapse rates, increasing treatment adherence, improving socio-occupational and family functioning, and reducing stress and family burden. When implemented optimally, it can be an effective tool that complements other treatment options, such as psychopharmacology and other psychosocial interventions.

**Objectives:**

To describe a clinical case of severe mental disorder, emphasizing the special relevance of a comprehensive approach.

**Methods:**

The case of a 46-year-old woman, with no prior mental health history, is described. She began follow-up after experiencing several psychotic episodes induced by substance use (cannabis). It was agreed with the patient to start psychopharmacological treatment and a psychotherapeutic approach by incorporating her into the center’s Multifamily Group (MFG). Initially, she received treatment with olanzapine 15 mg maximum daily dose (MDD), but it was discontinued due to the metabolic syndrome that appeared as a side effect. Treatment with lurasidone 74 mg MDD was then started, but it was also eventually discontinued due to intense akathisia. During the three years of follow-up, the patient remained abstinent from cannabis, without psychopharmacological treatment, and received intensive therapy in the MFG. Over time, her delusional ideation of persecution and self-referential clinical symptoms progressively subsided until resolution.

**Results:**

During a session of the MFG, in the context of several stressful situations (her son moving out of the family home, personal experience with the stigma of mental illness after a reading in the MFG, and frequent arguments with her husband), the patient reported delusional ideation and self-referentiality again, predominantly affecting her emotional state. In agreement with the patient and considering the side effects previously experienced with other antipsychotics, it was decided to initiate treatment with Brexpiprazole at progressively increasing doses until reaching 4 mg MDD, with good tolerability and clinical efficacy.

**Conclusions:**

This case highlights the particular importance of adopting a multimodal approach for the effective management of severe mental disorders, which can optimize clinical outcomes, promote more sustainable recovery, and improve the quality of life for patients.

**Disclosure of Interest:**

None Declared

